# Diverse temporal and spatial mechanisms work, partially through Stanniocalcin-1, V-ATPase and senescence, to activate the extracellular ATP-mediated drug resistance in human cancer cells

**DOI:** 10.3389/fonc.2024.1276092

**Published:** 2024-02-06

**Authors:** Haiyun Zhang, Jingwen Song, Ryan Ward, Yong Han, Arabella Hunt, Pratik Shriwas, Alexander Steed, Cory Edwards, Yanyang Cao, Milo Co, Xiaozhuo Chen

**Affiliations:** ^1^ Department of Biological Science, Ohio University, Athens, OH, United States; ^2^ The Edison Biotechnology Institute, Ohio University, Athens, OH, United States; ^3^ The Program of Molecular and Cellular Biology, Ohio University, Athens, OH, United States; ^4^ The Honor Tutorial College, Ohio University, Athens, OH, United States; ^5^ Heritage College of Osteopathic Medicine, Ohio University, Athens, OH, United States; ^6^ Department of Biomedical Science, Heritage College of Osteopathic Medicine, Ohio University, Athens, OH, United States

**Keywords:** EMT, cancer stem cells, cancer metabolism, transcriptomics, metabolomics, gene knockdown, lysosome

## Abstract

**Introduction:**

Resistance to drug therapies is associated with a large majority of cancer-related deaths. ATP-binding cassette (ABC) transporter-mediated drug efflux, epithelial-mesenchymal transition (EMT), cancer stem cells (CSCs), glutathione (GSH), senescence, and vacuole-type ATPase (V-ATPase) all contribute to the resistance. We recently showed that extracellular ATP (eATP) induces and regulates EMT, CSC formation, and ABC transporters in human cancer cells and tumors. eATP also consistently upregulates *Stanniocalcin-1 (STC1)*, a gene that significantly contributes to EMT, CSC formation, and tumor growth. We also found that eATP enhances drug resistance in cancer cells through eATP internalization mediated by macropinocytosis, leading to an elevation of intracellular ATP (iATP) levels, induction of EMT, and CSC formation. However, these factors have never been systematically investigated in the context of eATP-induced drug resistance.

**Methods:**

In this study, we hypothesized that eATP increases drug resistance via inducing ABC efflux, EMT, CSCs, *STC1*, and their accompanied processes such as GSH reducing activity, senescence, and V-ATPase. RNA sequencing, metabolomics, gene knockdown and knockout, and functional assays were performed to investigate these pathways and processes.

**Results and discussion:**

Our study results showed that, in multiple human cancer lines, eATP induced genes involved in drug resistance, elevated ABC transporters’ efflux activity of anticancer drugs; generated transcriptomic and metabolic profiles representing a drug resistant state; upregulated activities of GSH, senescence, and V-ATPase to promote drug resistance. Collectively, these newly found players shed light on the mechanisms of eATP-induced as well as *STC1*- and V-ATPase-mediated drug resistance and offer potential novel targets for combating drug resistance in cancers.

## 1 Introduction

Drug resistance contributes to up to 90% of all cancer related deaths (1). Drug resistance in cancer is a complicated response to cellular stress induced by cytotoxic compounds such as intracellular/extracellular metabolites, natural compounds (2, 3) and anticancer drugs (4, 5). Cancer cells hijack the response of normal cells to expel intracellularly located anticancer drugs and/or induce anti-cytotoxic reactions to cope with the drug-induced stress for increased survival capabilities. Cancer cells mobilize all the weapons available to them, including signaling and metabolic pathways, to fight against the detrimental reactions induced by the drugs. The diverse strategies used by cancer cells in generating drug resistance include activating plasma membrane-bound ATP-binding cassette (ABC) transporters to efflux toxic drugs or harmful metabolites (6, 7); utilizing glutathione (GSH) to moderate oxidative stress or inflammation to maintain redox homeostasis (8, 9); inducing senescence to exit the cell cycle and become quiescent cells for increased resistance to drug-induced apoptosis (10, 11). In addition, epithelial to mesenchymal transition (EMT), and cancer stem cells (CSCs) in the cancer cell population also play important roles in keeping cancer cells in tumors in a resistant state, further enhancing their survival in the presence of anticancer therapeutics (12–15). Different combinations of the pathways or processes in cancer cells are induced by different drugs depending upon the inhibitory properties or targets of the drugs, the cancer cell’s intracellular biological/metabolic conditions, and the tumor microenvironment (TME), and TME-cancer cell dynamic interactions. A better understanding of these mechanisms is imperative to more effectively combat drug resistance and improve therapeutic effectiveness.

Drug resistant status in cancer has been known to be closely associated with its intracellular ATP (iATP) levels. Cancer cells exhibit higher iATP levels than the normal cells from which they are derived (16). Resistant cancer cells selected from the treatment of anticancer drugs show even higher iATP levels than regular cancer cells (17, 18). Thus, drug resistance is correlated with and directly proportional to iATP levels. How and why such higher iATP levels are generated was not fully understood. The contributions of extracellular ATP (eATP) to iATP levels and drug resistance were largely unknown until we reported that eATP, which is present in tumors or the tumor microenvironment (TME) at concentrations that are at least 1000 times higher than those found in normal tissues (19–21), is internalized by cultured cancer cells and grafted human tumors by macropinocytosis (22–24). Macropinocytosis is an endocytic process closely associated with cancer metabolism and is significantly upregulated in almost all cancer cells (25, 26). The internalized eATP is then released by leaky macropinosomes (27, 28), mixed with the iATP pool, drastically elevates iATP levels, and enhances resistance to both target and chemotherapy drugs of various cancer cell lines from multiple cancer types (29–31). The increased resistance correlates with enhanced ABC transporter activities, phosphorylation status of key kinases, and reduced apoptosis (29). Our recent transcriptomics study revealed that 11 EMT-related genes were consistently and significantly upregulated by both eATP and TGF-β , the latter being a well-known EMT inducer that also induces heightened drug resistance (32, 33). We have also shown that one of the genes, *Stanniocalcin-1* (*STC1*), plays important roles in CSC formation such as inducing and upregulating expression of CSC surface markers, the CSC subpopulation, and augmenting mitochondria-mediated ATP synthesis (34). As an EMT and CSC inducer, it is conceivable that *STC1* also contributes to drug resistance.

Vacuolar-type ATPases (V-ATPases) have also been known to contribute to drug resistance. The multi-subunit V-ATPase is expressed widely in plasma and organelle membranes and plays a vital role in lysosomal acidification and maintaining pH homeostasis (35). Malfunctional V-ATPases are closely linked with cancer progression, as drug-resistant cancer cells show higher lysosomal amplification and V-ATPase expression on the plasma membrane (36). Moreover, inhibition of V-ATPase activity with bafilomycin A remarkably suppressed cancer proliferation and reversed drug resistance (37, 38).

With ample reported evidence, provided from our own previous studies and others, on the relationships between EMT, CSC formation, senescence, *STC1*, and V-ATPase and their connection to drug resistance cited above, in this study, we hypothesized that eATP induces drug resistance at transcriptional, translational, and metabolic levels using some or all the mechanisms and pathways described above. We used RNA sequencing, protein analysis, and metabolomics to investigate these changes. In addition, we also used siRNA knockdown (KD) and CRISPR Cas 9 knockout (KO) strategies combined with different functional assays to decipher the mechanisms used by genes induced by eATP in drug resistance in human lung cancer cells and in other cancer cell types. Specifically, we used human non-small cell lung cancer (NSCLC) A549 cells with the *STC1* gene knocked out to study *STC1* functions in drug resistance. The results of this study provide mechanistic insights on how eATP, *STC1*, and V-ATPase induce and regulate drug resistance and identify potential novel therapeutic targets for combating drug resistance in cancer.

## 2 Materials and methods

### 2.1 Cell lines and cell culture

Human NSCLC cell lines A549 (RRID: CVCL_0023) and H1299 cells (RRID: CVCL_0060), and human liver adenocarcinoma SK-Hep-1 cells (RRID: CVCL_0525) were purchased from American Type Culture Collection (ATCC). These cells were cultured in standard Dulbecco’s Modified Eagle Medium (DMEM with 25mM glucose) supplemented with 10% fetal bovine serum, 1% penicillin, and 50ug/ml streptomycin. All cells were grown in a humidified cell incubator with 5% CO_2_ at 37°C.

### 2.2 Efflux of ^3^H-labeled anticancer drugs

A549 and H1299 cells were seeded at 40,000 cells/well in a 24 well-plate. The next day, the cells were treated with ATP and/or ^3^H-paclitaxel (Moravek, CA) at a concentration of 1 μM and 0.125 μCi/well or ^3^H-gefitinib (Moravek) at a concentration of 10 μM 0.077 μCi/well for desired time at 37°C with 5% CO_2_. Before harvest, cells were washed with cold PBS once. Then 200 μL 0.1 mM NaOH was added into each well and the plate was shaken for 30 minutes at room temperature for cell lysis. Finally, the cell lysate from each well was transferred into a vial with 2 mL scintillation fluid, and the radioactivity in cell lysate was measured with a liquid LS 6500 Scintillation Counter (Beckman Coulter, Indianapolis IN).

### 2.3 RNA extraction and RT-qPCR

A549 cells were seeded at 200,000 cells/well in 60mm Petri dishes. The next day the cells were treated with ATP (0.5 mM) and TGF-β (10 ng/mL) for desired time at 37°C with 5% CO_2_. Total RNA from A549 cells was extracted by an RNA purification kit (Thermo Fisher Scientific, Waltham, MA) following the manufacturer’s instruction. Total RNA (1 μg) was reverse transcribed using a cDNA synthesis kit (Thermo Fisher). cDNA was amplified using SYBR Green qPCR Master Mix (Thermo Fisher) in CFX Connect Real-Time PCR Detection System (Bio-Rad, Hercules CA). The primers targeting ABCB1, ABCC1, ABCG2, and β-actin were purchased (KiCqStart™, MilliporeSigma, Burlington MA) and the sequences are listed in (**Supplementary Table 1**). Thermal cycle conditions were: 95°C, 10 min; (95°C, 15 s) × 40; 95°C, 15 s; 60°C, 30 s. The expression of targeted genes was normalized against β-actin and quantified using the 2^−ΔΔCt^ method.

### 2.4 RNA sequencing

RNAseq was performed as previously described (30). Data was stored in the Gene Expression Omnibus (GEO) database with an accession number: GSE160671. The differentially expressed genes (DEGs) were selected with log_2_ (fold change) ≥1 or log_2_ (fold change) ≤-1, and with p values < 0.05.

### 2.5 Protein analysis

Proteins were isolated from human lung A549 cells and human liver SK-Hep-1 cells treated with or without ATP for the desired time at 37°C with 5% CO_2_. Proteins were analyzed with western blots using appropriate primary antibodies: cofilin (Rabbit, 1:3000, CST, #5175), ABCB1 (Rabbit, 1:1000, CST, #12683), ABCC1 (Rabbit, 1:1000, CST, #14685), and ABCG2 (Rabbit, 1:1000, CST, #42078). Secondary antibody staining was completed with anti-rabbit IgG, HRP-linked antibody (Goat, 1:1000, CST, #7074). Cofilin, which is less affected by the eATP treatment, was used as a protein loading control. The signals were detected with Super Signal West Pico Chemiluminescent substrate (Thermo Fisher Scientific). The images were acquired, and the quantification of Western blots was performed by Odyssey Fc imaging system (LI-COR).

### 2.6 Metabolomics

Samples were prepared as previously described (32) and were analyzed at the Ohio State University Campus Chemical Instrumentation Center (CCIC) (32).

### 2.7 Cell viability assay

Cell viability assays were performed using resazurin as a colorimetric dye as previously described (39). Briefly, A549 cells were seeded at 5,000 cells/well in a 96 well-plate. The next day, the cells were treated with sunitinib, ATP and/or Bafilomycin A1 at two concentrations at 37°C with 5% CO_2_ for 24 hours. Resazurin (SigmaAldrich) was then added to cell culture medium and incubated at 37°C with 5% CO_2_ until the medium started showing pinkish color. Fluorescence intensity was then measured by Cytation 3 imaging reader (Biotek) at an excitation/emission of 560/590 nm.

### 2.8 iATP concentration assay

iATP levels were measured by a firefly luciferase based ATPlite Luminescence assay system (PerkinElmer) according to manufacturer’s instructions and the detailed steps were previously described (40). Briefly, A549 cells were seeded at 10,000 cells/well a 96 well-plate. The next day, the cells were treated with ATP and/or Bafilomycin A1 of two concentrations at 37°C with 5% CO_2_ for the desired time. Then the cell’s culture medium was aspirated, and the cells were washed twice with PBS. iATP levels were measured using an ATPlite luminescence ATP detection assay (PerkinElmer). The final luminescence intensity was detected by Cytation 3 imaging reader (Biotek). Relative iATP levels were normalized to the control group.

### 2.9 Intracellular GSH measurement

A549 cells were seeded at 400,000 cells/well in 60 mm Petri dishes and treated with ATP at various concentrations for the desired time at 37°C with 5% CO2. Before harvest, cells were washed with PBS (containing 1 mM N-Ethylmaleimide) twice for 1 minute and then scraped off the dish in cold 80% methanol. The cell extracts were then centrifuged at 4°C at 10000 g for 5 minutes and supernatants were collected. The supernatants were evaporated to dryness and then re-dissolved in H_2_O and measured by HPLC (Waters, Milford MA).

#### 2.9.1 GSH/GSSG ratio assay

A549 cells were seeded at 10,000 cells/well in a 96 well-plate treated with 0.5 mM ATP for desired time at 37°C with 5% CO_2._ Upon harvest, cells were washed with PBS and lysed in ice cold 1x Mammalian Lysis Buffer (ab179835, abcam, Waltham MA). The cell lysate was then deproteinized with trichloroacetic acid (TCA) and neutralized with NaHCO_3_. Then the GSH/GSSG ratio was measured using GSH/GSSG Ratio Detection Assay Kit II (Fluorometric - Green) (ab205811) following the supplier’s manual.

### 2.10 Senescence assays

The senescence (chromogenic) assay was performed as described (41). Briefly, after treatment, the cells were washed twice with PBS, fixed with a 2% formaldehyde/0.2% glutaraldehyde PBS solution, washed twice with PBS again and then incubated overnight with an X-gal staining solution (1 mg/mL buffered to pH 6.0). The cells were then washed with PBS and methanol and allowed to dry. Cells were examined under bright field microscopy at 20x magnification for pictures, cell counts were done at 40x magnification across a strip spanning each well and going through the center.

### 2.11 *STC1* gene knockout study

The procedure of *STC1* gene knockout was performed as described previously (34). Wildtype A549 cells or A549stc1ko cells were treated with or without anticancer drugs sunitinib, gefitinib, or paclitaxel in the presence or absence of 0.5 mM ATP, and then measured for their cell viability by the cell viability assay 24 hours after the treatments. Wildtype A549 cells or A549stc1ko cells were treated with 0.5 mM ATP and incubated for 0, 1, 2, and 6 hours and iATP levels were measured by the iATP concentration assay. A549stc1ko and A549 cells were treated with 0.5 mM ATP for 0, 2, 6, 24 hours, then protein expression of ABCB1, ABCC1 and ABCG2 proteins was measured by western blot analyses. Cofilin was used as a protein loading control for protein normalization.

#### 2.11.1 Fluorescence microscopy

Filamentous-actin or F-actin of wildtype A549 cells or A549stc1ko cells was stained with Fluorescent Phallotoxins to indicate changes in cell protrusion such as filopodia. The procedure was performed as described previously (40). Briefly, the cells were seeded overnight on glass coverslips placed in 24-well plates, then treated with or without ATP of three different concentrations and for desired time. Before staining, cells on coverslips were washed three times with PBS and fixed with 4% formaldehyde solution in PBS at room temperature for 10 minutes. Then the cells were washed with PBS three times and permeabilized with 0.1% Triton X-100 in PBS for 5 minutes. After washings with PBS three times, fixed cells were incubated with 150 nM Alexa Fluor™ 488 Phalloidin (Thermo Fisher Scientific) dissolved in PBS for 20 minutes. Next, cells were washed with PBS three times and stained with ProLong Gold Antifade Mountant (Thermo Fisher Scientific) to visualize and verify the location of the nucleus. Stained cells were examined and photographed using a Fluor Motorized DIC Polarization Phase Contrast Microscope (Zeiss AXIO Observer) at 400X magnification.

### 2.12 V-ATPase study

To determine V-ATPase’s role in drug resistance, A549 cells were treated with 20 μM sunitinib (a target drug) + 0.5 mM ATP and/or Bafilomycin A1 (100, 200 nM), an inhibitor of V-ATPase (37, 38), and incubated for 24 hours; cell viability/proliferation was measured by resazurin assay. After the contribution of V-ATPase in the drug resistance was shown, A549 cells were treated with 0.5 mM ATP and/or Bafilomycin A1 (100, 200 nM) and incubated for 2, 4, 6, and 8 hours and iATP levels were measured by an ATP uptake assay to assess V-ATPase’s role in elevating iATP levels.

#### 2.12.1 *ATP6V0C* gene KD

The KD small interference RNA (siRNA) for *ATP6V0C* and negative control (scrambled) siRNA were purchased from Qiagen. The target sequence for *ATP6V0C* siRNA is 5’-CTGCTTAAACAAAGCAGTATA-3’. siRNA transfection was performed as previously described (40) using Lipofectamine RNAiMAX transfection reagent (Thermo Fisher Scientific) according to the manufacturer’s instruction. The KD efficiency was determined by RT-qPCR and the primer (5’-CTGCTTAAACAAAGCAGTATA-3’) targeting *ATP6V0C* was purchased (KiCqStart™, SigmaAldrich). 24 hours after the transfection, cells were treated with sunitinib and/or ATP for 24 hours and measured for cell viability.

### 2.13 Statistical analysis

All experiments were performed in at least triplicates (N=3-6), depending upon assays, and were repeated at least once. Results of RT-qPCR were presented as mean ± standard error, and all other results were presented as mean ± standard deviation. Student’s t-tests were performed to evaluate the differences between the two groups, and nonparametric ANOVA was used when multiple groups were compared. P<0.05 was considered statistically significant. *P<0.05, **P<0.01 and ***P<0.001.

## 3 Results

### 3.1 eATP induced efflux of target and chemotherapy drugs

**Figure 1:**
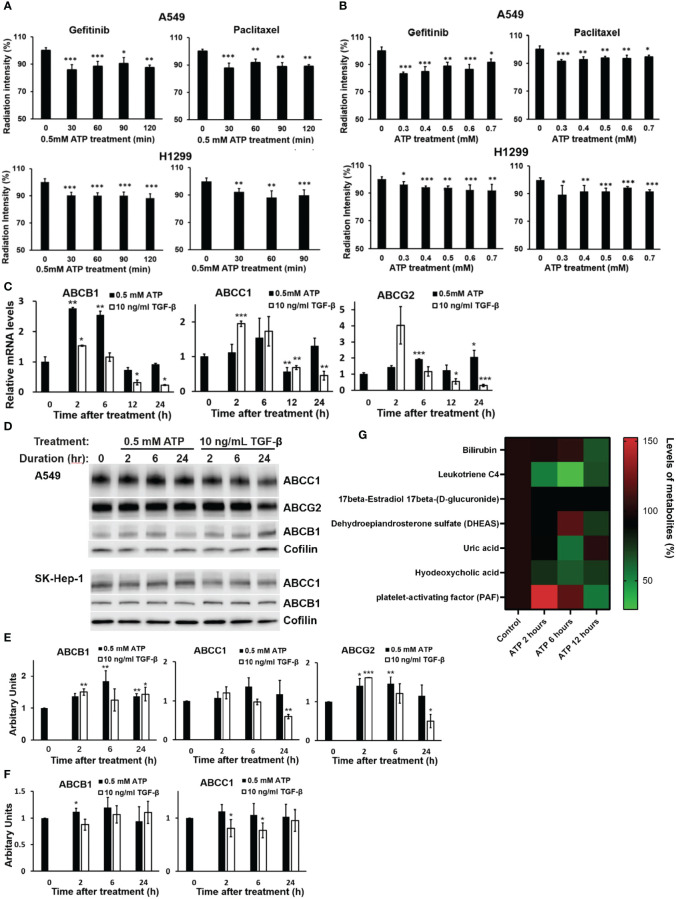
Extracellular ATP enhanced drug efflux and altered expression patterns of ABC genes and proteins. A549 and H1299 cells were treated with ^3^H-labeled gefitinib or ^3^H-labeled paclitaxel with or without for different times or different doses of ATP in A549 and H1299 NSCLC cell lines. The radiation intensity levels of intracellular ^3^H were measured with a scintillation counter. **(A)** Time course study: A549 and H1299 cells were treated with 0.5 mM ATP for various times. **(B)** ATP dose response study: A549 and H1299 cells were treated with various concentrations of ATP for 90 minutes. **(C)** mRNA levels of ABCB1 and ABCG2 detected by RT-qPCR in A549 cells. **(D)** Western blot analyses of ABC proteins in A549 (top) and SK-Hep1 (bottom) cells, and **(E)** Quantification of proteins ABCB1, ABCC1 and ABCG2 by western blots in A549 cells. **(F)** Detection and quantification of proteins ABCB1 and ABCC1 by western blot analysis in SK-Hep-1 cells. Cofilin was used as a protein loading control for protein normalization. **(G)** Extracellular ATP enhances efflux of physiological substrates of ABC transporters. A549 cells were treated with 0.5 mM ATP and incubated for 2, 6 and 12 hours, and levels of metabolites which are physiological substrates of ABC transporters were pulled out from metabolomics data file. A549 cells with no treatment serve as control. Transporters responsible for the efflux of different metabolites: bilirubin (ABCC2), leukotriene C4 (ABCC1, ABCC2), 17beta-Estradiol 17beta-(D-glucuronide) (ABCC1), DHEAS (ABCG2), uric acid (ABCG2), hyodeoxycholic acid (ABCC2), PAF (ABCB1). Data are presented as the mean ± standard deviation. Data of mRNA levels (Fig C) are presented as mean ± standard error; other data are presented as the mean ± standard deviation. Data of mRNA levels (Fig C) are presented as mean ± standard error; other data are presented as the mean ± standard deviation. *P < 0.05, **P < 0.01 and ***P < 0.001.

Drug efflux assays using approved radio-labeled drugs gefitinib and paclitaxel revealed that, in both A549 and H1299 NSCLC cells, eATP induced significant efflux of both drugs at the doses of eATP used and the times examined (**Figures 1A, B**). The reasons for using 0.5 mM ATP as a preferred ATP concentration are (i) this is the eATP concentration found in TME of several different tumor models (19–21) and (ii) it was the eATP concentration used in our previous and recent studies that show the best results in inducing drug resistance, EMT, and cancer stem cell formation (29–32, 34). eATP did not induce more drug efflux after 6 hours, not because it stopped the ABC’s efflux promoting activity but because a dynamic equilibrium between rates of drug efflux and influx is reached after 6 hours. These results are consistent with our previous observations that eATP induced resistance to these two drugs in these two cancer cell lines and the efflux of ABC type-specific dyes from these two cell lines (29, 34). It is worth noting that drug efflux profiles for the two drugs in the two cell lines are somewhat different, particularly in the trend of efflux over the eATP doses used (**Figure 1B**). The reason for the difference is presently unknown but could be related to the special properties of the two drugs and ABC transporters expressed in the two cell lines.

#### 3.1.1 eATP induced dose- and time-dependent changes in ABC mRNAs and proteins

To determine if eATP treatment alters expression of genes and proteins involved in the ABC system, qRT-PCR was performed, which showed that mRNA levels of ABCC1, ABCB1, and ABCG2, the ABC transporters responsible for efflux of gefitinib and paclitaxel in A549 cells, were significantly changed (mostly increased) over the times examined, particularly for the 2 and 6 hours (**Figure 1C**). ABC mRNA levels reduced approximately to basal levels at 12 and 24 hours, probably because increased levels of ABC proteins are not needed at these later times when the iATP levels in the eATP treated A549 cells are much higher than at 2 and 6 hours. At these times, enhanced ABC efflux activity is maintained by extra iATP as the energy for the ABC transporters, not by ABC mRNA/protein levels. At these later time points, a new dynamic equilibrium is achieved between ABC protein degradation and new ABC protein synthesis ~4-6 hours after the start of the experiment, so that the ABC protein levels are maintained at lower concentrations than at the beginning of the experiment when higher iATP levels are present due to ATP internalization by macropinocytosis. eATP induced higher increases in ABCB1 mRNA than TGF-β, while TGF-β induced higher increases in ABCC1 and ABCG2 than eATP after 2 hours (**Figure 1C**). Western blots showed that the protein levels of these ABCs (**Figure 1D**) were also changed similarly but not identically to those of the mRNAs. ABC protein changes similar to those found in A549 cells were also found in liver cancer SK-Hep-1 cells (**Figure 1D**). These indicate that the eATP induced ABC changes not only in NSCLC cells but also other cancer types. Quantifications of the mRNA and protein levels of select ABCs are shown in **Figures 1E, F**, respectively.

Metabolomics analysis of A549 cells treated with ATP for 2, 6, or 12 hours shows that physiological metabolic substrates for ABCs were pumped out significantly more than in the untreated control (**Figure 1G**), correlating with the increased ABC’s drug efflux activities (**Figures 1A, B**).

### 3.2 eATP induced changes in expression of genes involved in drug resistance

**Figure 2:**
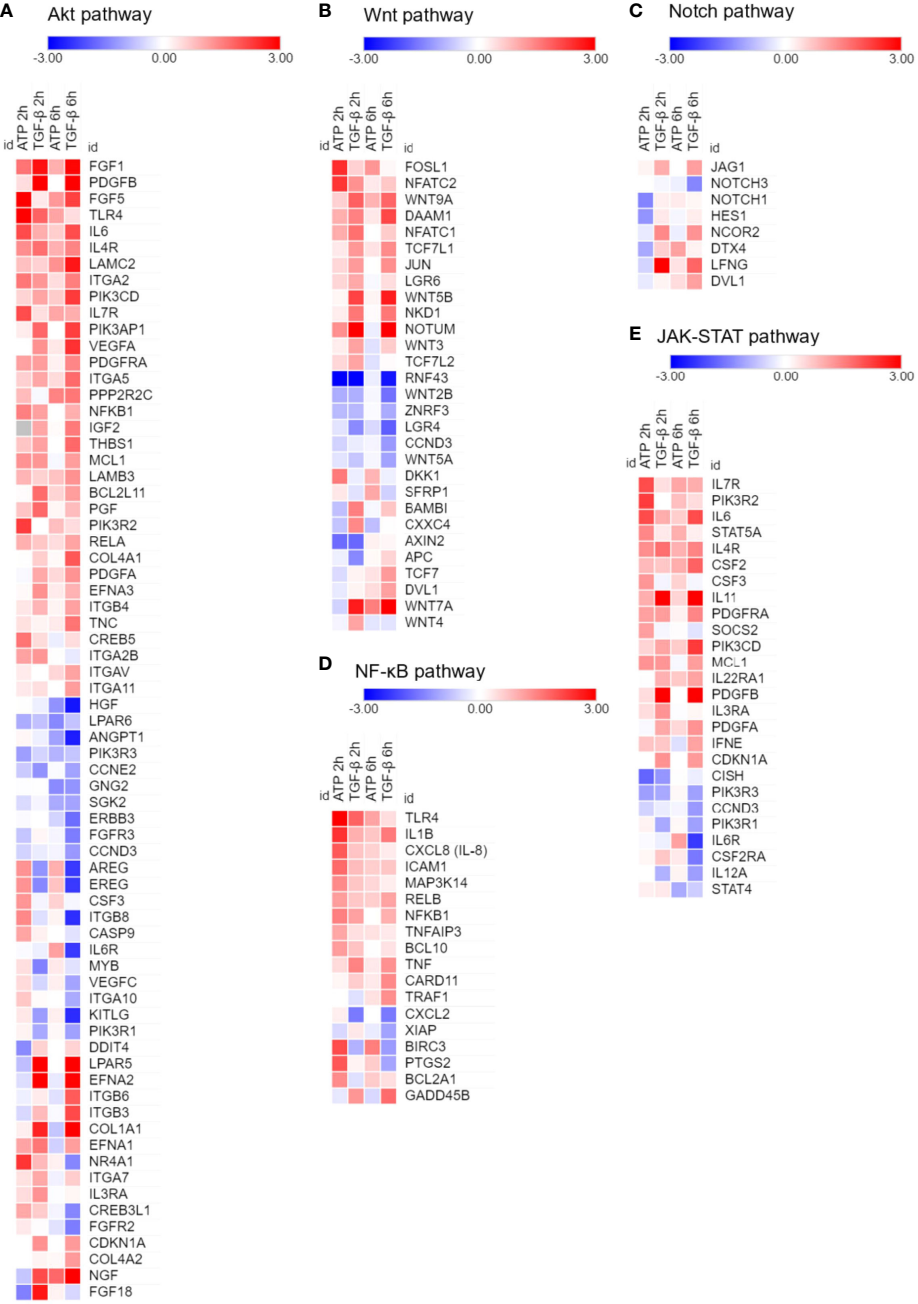
RNAseq analysis of A549 cells treated with eATP. A549 cells were treated with either ATP at 0.5 mM or TGF-β at 10 ng/mL for 0, 2 and 6 hours. Total polyA RNAs of treated cells were isolated and analyzed by RNAseq. Log2 (Fold change) value is used for the value bars in the heatmap. TGF-β served as gene expression controls. Gene heatmaps involved in different pathways: **(A)** Akt. **(B)** Wnt. **(C)** Notch. **(D)** NF-κB. **(E)** JAK-STAT.

RNA sequencing analysis was performed to examine the effects of eATP on the expression of genes involved in drug resistance-related non-ABC pathways and processes. These pathways and processes include AKT, Wnt, Notch, NF-κB, and JK-STAT (**Figures 2A-E**). TGF-β was used as a control for comparison. Among these pathways and processes, many genes were expressed in the same patterns in both eATP and TGF-β treatments. For example, at both 2 hours and 6 hours, in the Wnt pathway: *FosL1*, *Wnt9A*, *Wnt5B*, and *Wnt3* were upregulated in both treatments (**Figure 2B**). In the NF-κB pathway: *TLR4, IL1B, ICAM1, NFKB1*, and *TNF* were upregulated (**Figure 2D**). In the JAK-STAT pathway: *IL6, IL 11, IL4R, IL7R, PDGFB* and *PDGFA*, and *PDGFRA*, were upregulated, while *PIK3R1, PIK3R3*, and *CISH* were downregulated in both treatments (**Figure 2E**). In contrast, some other genes are expressed in opposite ways: higher in the eATP treatment, and lower in the TGF-β treatment, or vice versa. It is conceivable that ATP, functioning as an energy molecule, a transcription cofactor, and a phosphorylation donor, can induce the drug resistant state in ways either similarly or differently from those of TGF-β depending upon the exact role ATP plays in that specific pathway or process. TGF-β and eATP share purinergic receptor signaling, but TGF-β binds and activates TGF-βR as a unique mechanism. More specifically, the overall regulation of certain specific genes involved in a pathway, potentially due to differences in cross talks between these pathways and other differently regulated signaling pathways, can be mediated differently in eATP or TGF-β induced cells, leading to different expression levels of the same genes in cells treated with different molecules. Collectively, these results suggest that eATP induced a transcriptomic profile similar but nonidentical to that of TGF-β representing a drug resistant state at the gene expression level.

### 3.3 eATP induced increase in GSH levels

**Figure 3:**
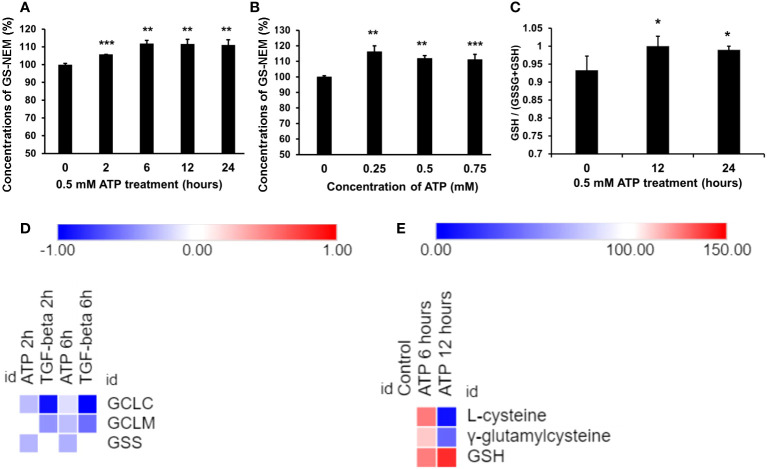
Extracellular ATP increased intracellular GSH levels. A549 cells were treated with various doses of ATP or incubated for various times, then intracellular GSH levels were measured with HPLC. *P < 0.05, **P < 0.01 and ***P < 0.001. **(A)** Time response study. A549 cells were treated with 0.5 mM ATP for different times, and intracellular GSH levels were measured and indicated by levels of N-ethyl succinimide-S-glutathione (GS-NEM). **(B)** Dose response study. A549 cells were treated with different concentrations of ATP and incubated for 6 hours. **(C)** A549 cells were treated with 0.5 mM ATP and incubated various times, the ratio of intracellular GSH over total GSH (GSH + GSSG) was detected by a GSH/GSSG Ratio Detection Assay Kit. **(D)** Heatmap of expression of genes involved in the GSH synthesis pathway. Log2 (Fold change) value is used for the value bars in the heatmap. **(E)** Heatmap of levels of metabolites involved in the GSH synthesis pathway. Relative levels of metabolites (100%) are used for the value bars in the heatmap.

GSH assays revealed that GSH was upregulated by eATP in a time course study (**Figure 3A**) and an eATP dose response study (**Figure 3B**). The study showed that it took more than 2 hours but fewer than 6 hours for GSH to reach its peak level. The ratio of (GSH/(GSSG+GSH) was also measured, and the result showed that the ratio increased compared with the control (**Figure 3C**), indicating a reduced cellular redox state. These results are consistent with the notion that eATP mediates oxidative stress and ROS levels through, at least in part, upregulating GSH levels.

RNAseq analysis revealed that the expression of the genes encoding the enzymes involved in the last two steps of synthetic pathway of GSH (**Figure 3D**), *glutamate cysteine synthase (GCLC and GCLM, two subunits of GCL)* and *glutathione synthetase* (*GSC)*, was slightly reduced but not to level of statistical significance (**Figure 3D**). However, the substrates and the intermediate products of these reactions, L-glycine and γ-glutamylcysteine, increased at 6 hours and decreased at 12 hours (**Figure 3E**). The final product GSH increased at both time points (**Figure 3E**). These results strongly suggest that these metabolic changes were via iATP elevation.

### 3.4 eATP induced senescent cells

**Figure 4:**
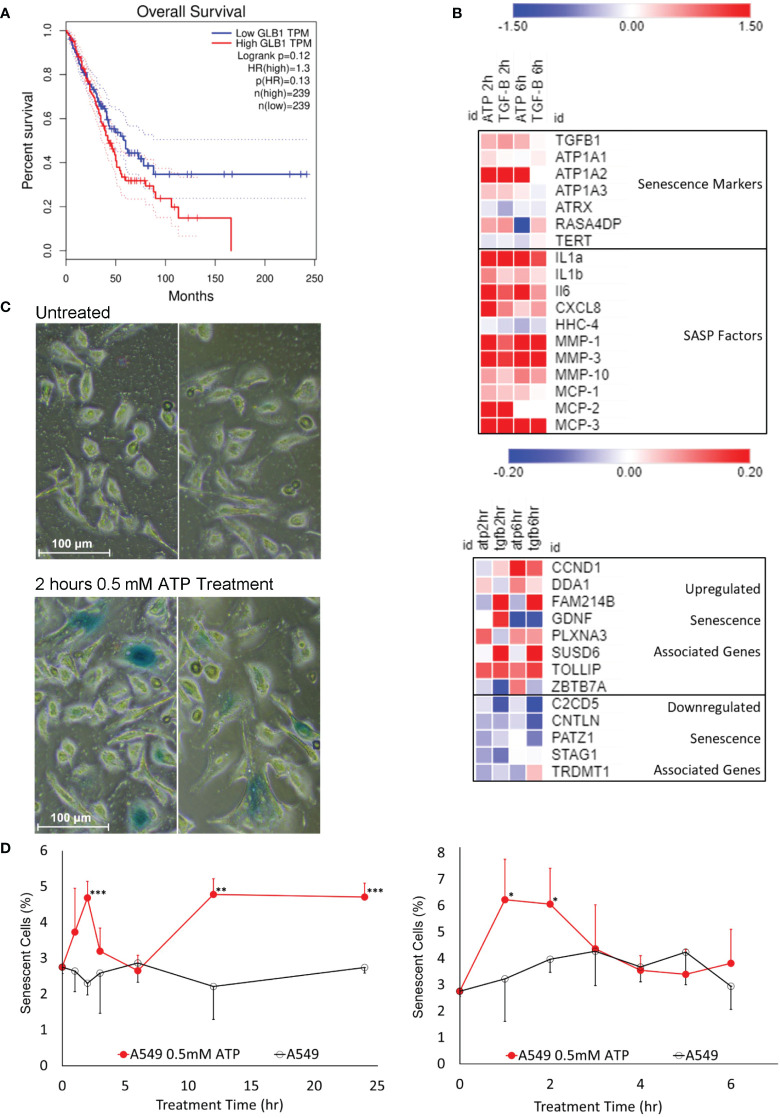
Senescence study: eATP induced senescence. A549 cells were treated with eATP at various concentrations for various times. The treated cells were analyzed by various assays. *P < 0.05, **P < 0.01 and ***P < 0.001. **(A)** Overall survival rates of high and low GLB1-expressing human lung cancer patients. Higher *STC1* levels were associated with a lower survival rate. Data was generated from GEPIA (http://gepia.cancer-pku.cn/index.html). **(B)** Heatmap of RNA sequencing data of senescence associated genes from various functional pathway categories. Red color represents gene upregulation and blue for downregulation. Names on the right side of the map are names of senescence related genes and categories. **(C)** eATP induces senescence in A549 cells. Human NSCLC A549 cells were treated without (top) or with (bottom) 0.5 mM ATP for 2 hours. Cells were stained with the senescence associated β-galactosidase activity chromogenic assay. Senescent cells stain blue. Senescent cells also generally appear larger than normal cancer cells. **(D)** Time course studies of A549 cells treated with or without 0.5 mM ATP.

Senescence is known to be involved in drug resistance in cancer (10, 11). To find out if eATP induces senescence, various assays were performed. β-Galactosidase (β-Gal) is known to be closely associated with senescence and its expressed levels are negatively correlated with survival of lung cancer patients (**Figure 4A**). RNAseq analysis showed that some of the genes involved in senescence (42) were significantly altered in the eATP treated cells, similar to those induced with TGF-β, a known inducer for drug resistance and senescence (**Figure 4B**). X-gal assay revealed that eATP treatment increased formation of senescent (bluish) cells (**Figure 4C**), as early as 1 to 2 hours and as long as 24 hours (**Figure 4D**). The temporary decline of induced senescence before 6 hour was correlated with our previous observation of the temporary decline of some EMT and CSC related genes, such as Sox2, Oct4, and Twist1 (34). Collectively, these results demonstrate that eATP induced senescence in A549 cells, and therefore drug resistance, as senescence is well-known to contribute to drug resistance.

### 3.5 Knockout study: *STC1* contributes to drug resistance

**Figure 5:**
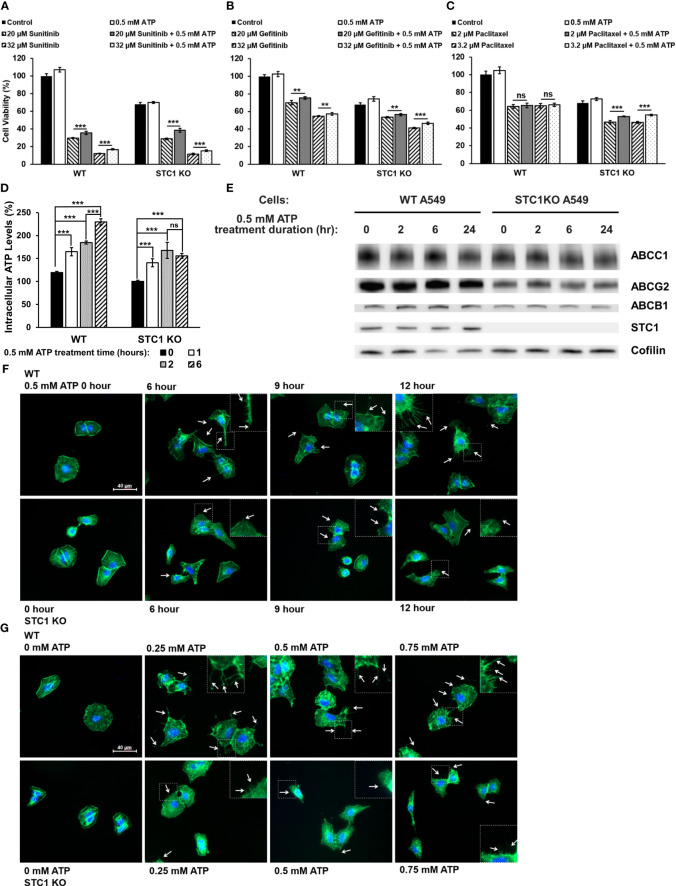
*STC1* knockout study Wildtype A549 cells or A549stc1ko cells were treated with or without anticancer drugs sunitinib, gefitinib, or paclitaxel in the presence or absence of 0.5 mM ATP, and then measured for their cell viability using resazurin assay 24 hr after the treatments. **P < 0.01 and ***P < 0.001. A549 cells were treated with **(A)** sunitinib. **(B)** gefitinib. **(C)** paclitaxel. **(D)** A549stc1ko and A549 cells were treated with 0.5 mM ATP and incubated for 0, 1, 2, and 6 hours, and iATP levels were measured. **(E)** A549stc1ko and A549 cells were treated with 0.5 mM ATP for 0, 2, 6, 24 hours, then protein expression of ABCB1, ABCC1 and ABCG2 proteins was measured by western blot analyses. Cofilin was used as a protein loading control for protein normalization. **(F, G)** eATP enhanced filopodia formation, while filopodia formation was reduced in A549stc1ko cells. Some of the cell images are enlarged for better viewing of the sizes and lengths of the filopodia formed. **(F)** Time response study: A549 and A549stc1ko cells were treated with 0.5 mM ATP and incubated for 0, 6, 9, and 12 hours. **(G)** Dose response study: A549 and A549stc1ko cells were treated with ATP of different concentrations and incubated for 6 hours. In both **(F, G)**, filopodia were shown as the projections on cell surfaces stained with green fluorescence (indicated by white arrows), and cell nuclei were stained with blue fluorescence (DAPI). ns, not significant.

We recently reported that the protein *STC1* significantly contributes to CSC formation and tumor growth (34). In this study, it was found that in both A549 and A549stc1ko cells, the presence of 0.5 mM ATP significantly increased the viability of the cells treated with sunitinib (**Figure 5A**) or gefitinib (**Figure 5B**). In contrast, eATP did not rescue A549 cells treated with chemo drug paclitaxel but partially and significantly rescued A549stc1ko1 cells (**Figure 5C**). These results suggest that, since sunitinib and gefitinib are competitive binders of ATP binding sites of protein kinases and their anticancer activities are related to and inversely proportional to iATP concentration, thus eATP could rescue their treated cancer cells by enhancing iATP levels through eATP internalization and therefore drug resistance. In contrast, paclitaxel is a chemotherapy drug with an anticancer activity unrelated to ATP. Thus, eATP did not increase the viability of paclitaxel treated A549 cells (**Figure 5C**). However, eATP did increase the viability of A549stc1ko cells treated by paclitaxel (**Figure 5C**). This is due to the fact that part of the *STC1* function is in increasing mitochondrial ATP synthesis (32). Therefore, the deletion of the *STC1* gene in A549stc1ko cells resulted in reduction in iATP concentration. eATP treatment led to large increases of iATP level at 1, 2, and 6 hours. (**Figure 5D**), but the iATP increase was much smaller in A549stc1ko cells than in A549 cells (**Figure 5D**). A protein analysis revealed that A549stc1ko cells show much lower protein levels for ABCC1, ABCG2, and ABCB1 compared with those of A549 cells (**Figure 5E**, quantified in **Supplementary Figure 1**). Fluorescence microscopy studies showed that eATP induced more filopodia formation and the filopodia were longer in A549 cells than those found in A549stc1ko cells over time (**Figure 5F**) or over different doses of ATP (**Figure 5G**). These results suggest that *STC1* affects filopodia formation, a process involved in cytoskeleton remodeling and EMT (34, 40). Since cytoskeleton remodeling and EMT play roles in drug resistance in cancer cells, it can be inferred that *STC1* also plays roles in the drug resistance.

### 3.6 V-ATPase studies

**Figure 6:**
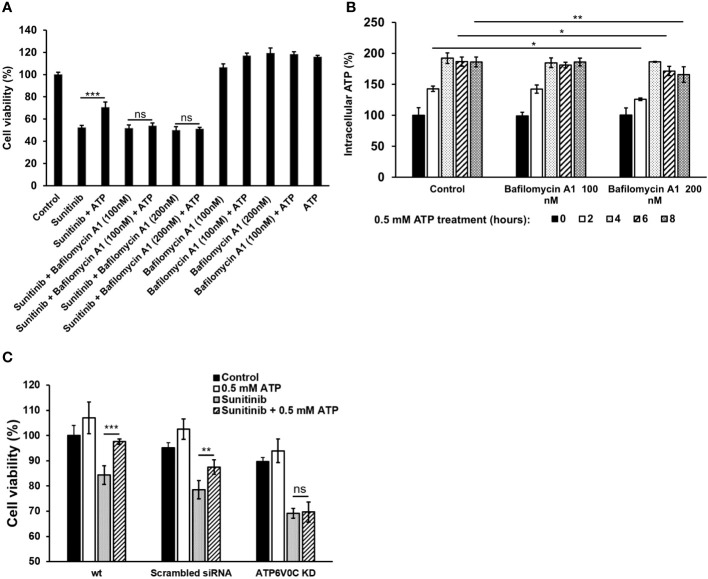
V-ATPase study A549 cells were treated with anticancer drug sunitinib and 0.5 mM ATP. Various concentrations of Bafilomycin A1, an inhibitor of V-ATPase, were added to the treated cells. Resazurin cell viability and ATP assays were performed. *P < 0.05, **P < 0.01 and ***P < 0.001. **(A)** Dose response of cell viability assay for bafilomycin: Different amounts of Bafilomycin A1 were added. **(B)** iATP concentration time course study of the Bafilomycin A1 inhibition assay. **(C)** siRNA knockdown of V-ATPase and cell viability was measured. ns, not significant.

To investigate the potential role of V-ATPase in the drug resistance, a V-ATPase small molecule inhibitor, bafilomycin A1 (37, 38), was used. Although bafilomycin A1 did not further reduce sunitinib-induced cell viability decrease, it inhibited the viability increase induced by ATP (**Figure 6A**). This result suggests that bafilomycin A1’s function here is to interfere with eATP’s activity, probably by interfering eATP internalization or the release of the internalized eATP from macropinosomes or endosomes/lysosomes, as V-ATPase is highly expressed in these endosomes. An iATP concentration assay demonstrated that the addition of bafilomycin A1, particularly at 200 nM, significantly reduced the iATP levels (**Figure 6B**). This result is consistent with those found in **Figure 6A**, suggesting that the reason bafilomycin inhibited the ATP’s viability-increase activity is due to, at least in part, its iATP-decreasing activity.The V-ATPase activity was also examined in a KD study, in which a gene encoding a specific subunit of V-ATPase, *ATP6V0C* (35, 36), was knocked down (KD). The study results show that eATP partially restored iATP level decreased by sunitinib in A549 cells without siRNA or with scrambled siRNA treatment. However, eATP did not restore iATP levels in A549 cells treated with *ATP6V0C*-specific siRNA (**Figure 6C**). This result is consistent with those in **Figures 6A, B**, suggesting that V-ATPase, *ATP6V0C* specifically, contributes to drug resistance by maintaining or increasing iATP levels.

### 3.7 Model for eATP induced drug resistance in human lung cancer

**Figure 7:**
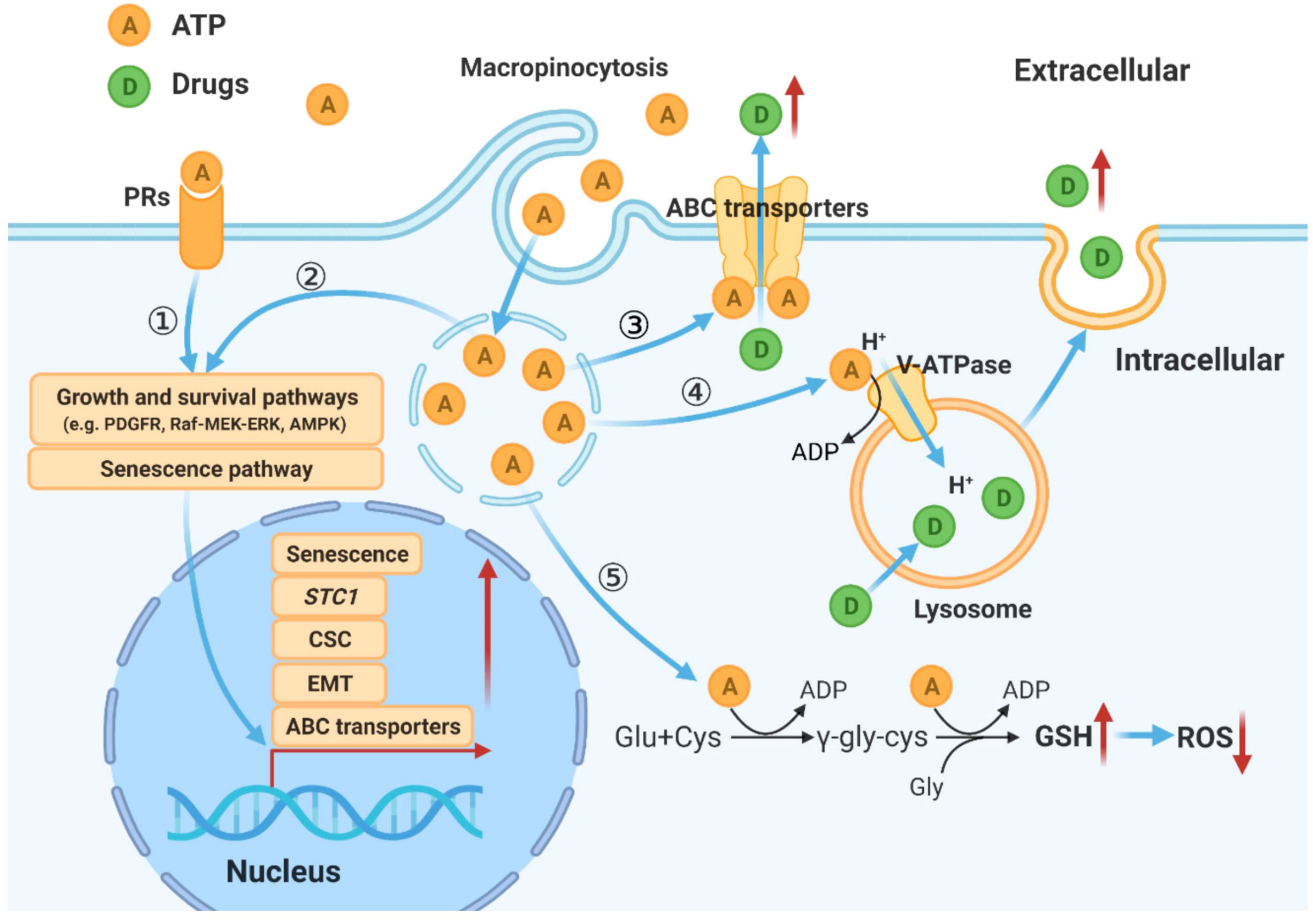
Hypothetical model for mechanisms by which eATP temporally and spatially regulates drug resistance in cancer cells. eATP works from outside of cancer cells as a messenger, binding to and activating purinergic receptors, and triggering EMT, and other functional changes of cancer cells (27–29). eATP is also internalized by enhanced macropinocytosis, entering cells and released by leaky macropinosomes, greatly increasing iATP levels (21, 22). The augmented iATP then functions as an energy molecule to accelerate biochemical reactions (21, 22, 30), a phosphorylating molecule to enhance signal transduction (21, 22, 32), and a transcription cofactor to speed up gene expression (30, 32). These mechanisms work together to alter/increase ABC transporter activity, GSH levels, senescence, V-ATPase activities, and anti-apoptosis, leading to an enhanced drug resistant state.

Based on studies described above combined with our and others’ previous studies, a hypothetical model for eATP induced drug resistance is proposed (**Figure 7**).

## 4 Discussion

Drug resistance in cancer is a multi-faceted and multi-pathway process through which cancer cells evade the cytotoxic effects of anticancer therapeutics. Our study demonstrates that eATP activated all the pathways and processes in multiple cancer types we examined to increase drug resistance and cancer cell survival. Thus, eATP is a general inducer of drug resistance in cancer.

In this study, we showed that eATP enhanced efflux of gefitinib and paclitaxel, an approved target drugs and a chemotherapy drug, respectively, in A549 and H1299 NSCLC cells (**Figure 1**). Although we previously showed that eATP triggered an increased efflux of an ABCB1- and ABCC1-dependent dye in A549 cells (29), this is the first time that real anticancer drugs were directly shown to be pumped out by eATP-treated in two NSCLC cell lines (**Figure 1**). This efflux activity is likely via ABCB1 and ABCC1 transporters since these ABC transporters and their efflux activity in these two cell lines were previously shown to be upregulated by eATP (29). In addition, the upregulation of ABC transporters in these two cell lines by eATP was directly demonstrated (**Figure 1**). Furthermore, we showed that eATP reduced retention of several metabolites, which are physiological substrates of ABCB1, ABCG2, or ABCC1 (**Figure 1**). All these results indicated that, although ABCC1 members showed little change in expression under eATP treatment (**Figure 1**), their efflux activity was indeed enhanced by eATP. This result is consistent with all other results, which show that the increased efflux is primarily due to enhanced efflux rate, not by ABC protein level increases. In all these cases, eATP is likely to be first internalized by macropinocytosis, as we showed before (22–24), and then released from macropinosomes, elevating iATP levels and then enhancing the efflux activity by functioning as a source of extra energy for ABCs specifically expressed in human NSCLC cells.

In our previous transcriptomic study, we found that the *STC1* gene significantly contributes to the induction of EMT and formation of CSCs in A549 cells (32, 34). Since both EMT and CSCs are intimately involved in drug resistance in cancer (12–15), we speculated that *STC1* also participates in the drug resistance. Our present KD and KO studies validate our hypothesis that *STC1* significantly contributes to drug resistance, mostly likely through its ATP synthesis-promotion, iATP-elevating activity, and filopodia formation-activating and thus EMT-promoting activity (34). Thus, *STC1* is likely to contribute to drug resistance by providing more ATP for the ABC drug efflux activity and accelerating biochemical reactions.

GSH is known to scavenge excessive ROS, detoxify detrimental xenobiotics (8), and thus protect cancer cells from oxidative stress induced by ROS-based therapy such as chemotherapy (8, 9). Thus, the identified increased cellular GSH levels by eATP (**Figure 3**) are likely to contribute to eATP-mediated resistance. These results suggest a robust ROS scavenging system, which is a known feature of CSCs for the purpose of maintaining low ROS levels and thus sustaining stemness properties (8). These results are also consistent with our previous finding that eATP promotes CSC induction (34) and thus likely contribute to CSC-related resistance.

Senescence is also an integral component of drug resistance. Although senescence can work as a suppressive mechanism which stops cell proliferation and prevents propagation of malignant cells, it can also lead to resistance to apoptosis in cancer cells induced by drug therapies (10, 11). The gene expression of β-Gal, the most prominent marker of senescence, is highly correlated with the survival of lung cancer patients (**Figure 4A**). eATP treatment resulted in increased levels of numerous genes involved in various aspects of senescence (**Figure 4B**) and number of senescent cells in A549 cells (**Figure 4C**) in a time-dependent manner (**Figure 4D**). Thus, it is concluded that eATP induces senescence and therefore drug resistance. Notably, this is the first time that eATP is shown to induce senescence in cancer cells.

V-ATPases express widely in plasma and organelle membranes and play a vital role in lysosomal acidification and maintaining pH homeostasis (35). Moreover, inhibition of V-ATPase activity with bafilomycin A remarkably suppressed cancer proliferation and reversed drug resistance (37, 38). Our study using both bafilomycin A1 and siRNA to inhibit V-ATPase confirms this result in lung cancer cells (**Figure 6**). Furthermore, V-ATPase inhibition abolished the eATP-induced cell rescue from anticancer drug treatment (**Figure 6**). These results verify that V-ATPase is a key player in eATP-induced drug resistance.

V-ATPase is involved in the regulation of macropinocytosis by maintaining lysosomal acidification (43). High V-ATPase activity is also tightly related to EMT and metastasis (43). Another of mechanisms of drug resistance induced by enhanced V-ATPase activity is that more drugs are taken into lysosome by ABC transporters located in the lysosomal membrane and released into extracellular environment (33). Our present study is consistent with these results, indicating that V-ATPase is involved in eATP-induced drug resistance in lung cancer.

Based on our previous and present studies, and study results reported by others, we propose a hypothetical model for eATP’s overall activities in drug resistance in cancer. In this model, eATP induces and regulates drug resistance spatially and temporally through multiple pathways and processes. First, eATP induces drug resistance extracellularly by binding to and activating purinergic receptors (PRs) located on the plasma membrane of cancer cells (29–31, 44, 45). The activated PRs (e.g. P2X7) send activating signals eventually to the nucleus to activate the expression of genes related to EMT, CSC formation, and senescence, three processes which greatly contribute to drug resistance (10, 11, 29–31, 40, 44–46). eATP also functions intracellularly. eATP is internalized primarily by commonly upregulated macropinocytosis and other minor endocytic processes by cancer cells (22–26) and released by leaky macropinosomes (27, 28), greatly elevating iATP levels (22, 23). The increased iATP in turn further enhances the efflux activity of plasma membrane-associated ABC transporters by providing more ATP as required energy (29–31). The elevated iATP also functions as a phosphate donor, augmenting protein phosphorylation of factors involved in signal transduction of pathways for cell survival and senescence (10, 11, 47, 48), and enhancing GSH production to mediate ROS levels. Once the elevated iATP reaches nucleus, it acts as cofactors for transcription, working together with the PR signaling, to trigger and regulate expression of genes involved in EMT and CSC formation, and moving cancer cells towards the formation of a drug resistance state (32, 34, 49, 50).

Temporally, some eATP activities, such as PR activation, are induced in seconds. Other short-term activities, such as iATP level elevation, and increased ABC efflux (**Figures 5D**, **1A**) are observed in minutes in both cancer cells (22) and tumors (23, 24). eATP-induced expression of genes related to EMT and/or CSCs are detected in no more than 2 hours (**Figures 1C**, **2**) (32, 34), EMT and CSC-related proteins markers in 2 to 6 hours (**Figure 1E**) (32, 34), and a drug resistant (EMT and CSC) state in 6 hours (34). eATP also induces GSH activity within 2 hours (**Figure 3**). Early onset of senescence is also induced within 2 to 6 hours (**Figure 4**). This is consistent to the observation made by others in rat liver for what they defined as “acute senescence” (51) In addition, import of drugs into lysosomal compartments (52) and the decrease in ROS levels [**Figure 3C**, (53)] also contribute to the final drug resistance. Thus, some observed activities are almost simultaneously induced and others sequentially. These induced activities last more than 24 hours, the longest measurement time in our study. Importantly, eATP is capable of inducing all these EMT/CSC/drug resistance-required activities, just like the well-established inducer TGF-β (54, 55). Thus, eATP functions as a master inducer and regulator for these coordinated activities. These activities are likely to be induced for multiple purposes beyond just drug resistance.

At this moment, it is unclear if all these induced activities are required for the drug resistance and what the functional relationships among these activities relative to drug resistance are. Furthermore, our studies have been focused on the early stage of the ATP induced drug resistance. It remains to be seen if the drug resistance induced by ATP is as long lasting as TGF-β, and if the mechanisms used at the later stage of the drug resistance are the same of the early stage. Additional studies are required to finally and fully validate the model.

Collectively, we showed that eATP induces and regulates drug resistance in human lung cancer cells by using multiple mechanisms. These eATP-induced and regulated mechanisms include ABC transporters, ROS/GSH response, senescence, and lysosomal V-ATPase. All these mechanisms are known to be related to EMT and CSC. Also, the inductions/regulation by eATP are at transcriptional, translational, and metabolic levels. For the first time, we show that GSH, *STC1*, senescence, and V-ATPase are involved in eATP-induced drug resistance. These new findings immediately identify new targets for combating drug resistance in cancer. Interfering eATP’s and iATP’s activities is likely to be very effective (56, 57), as it simultaneously activates multiple and essential drug resistance mechanisms (58).

## Data availability statement

The datasets presented in this study can be found in online repositories. The names of the repository/repositories and accession number(s) can be found below: https://www.ncbi.nlm.nih.gov/geo/, GSE160671.

## Ethics statement

Ethics approval is not required for either humans or animals for this study because only commercially available human cancer cell lines were used.

## Author contributions

HZ: Funding acquisition, Investigation, Methodology, Writing – original draft, Writing – review & editing, Conceptualization, Formal analysis, Validation. JS: Investigation, Methodology, Writing – review & editing. RW: Investigation, Methodology, Writing – original draft, Writing – review & editing, Formal analysis, Validation. YH: Methodology, Writing – review & editing. AH: Methodology, Writing – review & editing. PS: Methodology, Writing – review & editing. AS: Investigation, Writing – review & editing. CE: Investigation, Writing – review & editing. YC: Methodology, Writing – review & editing. MC: Writing – review & editing. XC: Funding acquisition, Investigation, Methodology, Resources, Supervision, Writing – original draft, Writing – review & editing, Conceptualization.
