# Supplementary material for: Diverse temporal and spatial mechanisms work, partially through Stanniocalcin-1, V-ATPase and senescence, to activate the extracellular ATP-mediated drug resistance in human cancer cells
Source: Front Oncol. 2024 Feb 6;14:1276092. doi: 10.3389/fonc.2024.1276092 (PMC10876858; doi:10.3389/fonc.2024.1276092)
Supplement: Supplementary file 1 [file Table_1.docx]

| Gene | Primer sequence |
| --- | --- |
| ABCB1 | Forward:  5′-AGTGAAAAGGTTGTCCAAG-3′ |
|  | Reverse:  5′-AGTCTGCATTCTGGATGG-3′ |
| ABCC1 | Forward:  5′-AGCAGAAAAATGTGTTAGGG-3′ |
|  | Reverse:  5′-TACCCACTGGTAATACTTGG-3′ |
| ABCG2 | Forward:  5′-AAAGCCACAGAGATCATAGAG-3′ |
|  | Reverse:  5′-GATCTTCTTCTTCTTCTCACC-3′ |
| β-actin | Forward:  5′-GACGACATGGAGAAAATCTG-3′ |
|  | Reverse:  5′-ATGATCTGGGTCATCTTCTC-3′ |

**Supplementary Table 1**. Primer sequences used for RT-qPCR.
