# Supplementary figures and images for: Diverse temporal and spatial mechanisms work, partially through Stanniocalcin-1, V-ATPase and senescence, to activate the extracellular ATP-mediated drug resistance in human cancer cells
Source: Front Oncol. 2024 Feb 6;14:1276092. doi: 10.3389/fonc.2024.1276092 (PMC10876858; doi:10.3389/fonc.2024.1276092)

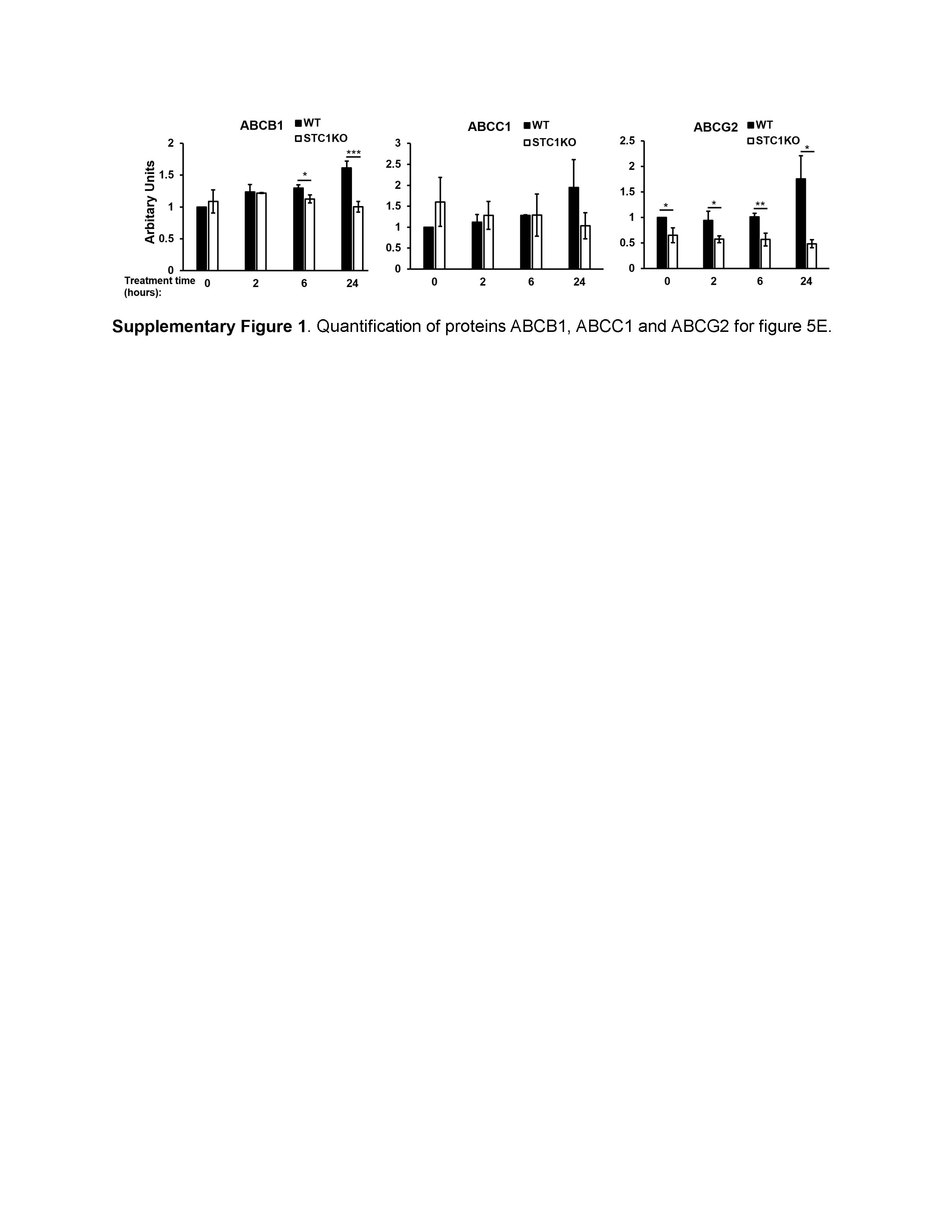

Supplement: Supplementary file 2 [file Image_1.jpeg]
